# A PET imaging study of the brain changes of glucose metabolism in patients with temporal lobe epilepsy and depressive disorder

**DOI:** 10.1186/s12880-021-00547-x

**Published:** 2021-02-22

**Authors:** Jin-Feng Wen, Xin-Wen Guo, Xiang-Yi Cao, Ji-Wu Liao, Ping Ma, Xiang-Shu Hu, Ji-Yang Pan

**Affiliations:** 1 Department of Psychiatry, Guangdong, The First Affiliated Hospital of Jinan University, No.613, West Huangpu Avenue, Tianhe District, Guangzhou, 510630 China; 2 Department of Psychology and Behavior, Guangdong Sanjiu Brain Hospital, Guangzhou, 510510 China; 3 Epilepsy Center, Guangdong Sanjiu Brain Hospital, Guangzhou, 510510 China

**Keywords:** Epilepsy, Temporal lobe, Depressive disorder, PET imaging, Glucose metabolism

## Abstract

**Background:**

This study aims to compare the difference of the brain changes of glucose metabolism between temporal lobe epilepsy patients (TLE) with major depressive disorder and temporal TLE without major depressive disorder.

**Methods:**

A total of 24 TLE patients, who met the inclusion criteria of our hospital, were enrolled in this study. They were divided into a TLE with depression group (n = 11) and a TLE without depression group (n = 13), according to the results of the HAMD-24 Scale. Two groups patients were examined using ^18^F-FDG PET brain imaging.

**Results:**

The low metabolic regions of the TLE with depression group were mainly found in the left frontal lobe, temporal lobe and fusiform gyrus, while the high metabolic regions of the TLE with depression group were mainly located in the right frontal lobe, visual joint cortex and superior posterior cingulate cortex. Both of the TLE groups had high metabolic compensation in the non-epileptic area during the interictal period.

**Conclusions:**

There is an uptake difference of ^18^F-FDG between TLE patients with depression and TLE patients without depression in multiple encephalic regions.

## Background

Temporal lobe epilepsy (TLE) originates from the hippocampus, the amygdala, the parahippocampal gyrus and the neocortex of the lateral temporal lobe [1]. The characteristics of TLE go far beyond seizures. Many patients develop special forms of cognitive impairment and psychiatric comorbidity [2]. Depressive disorder is the most common complication of TLE. However, comorbid depressive disorder in epileptic patients is often ignored, and only a few patients receive antidepressant treatment [3]. Hence, it is of great clinical significance to study the diagnosis, treatment and pathogenesis of TLE with depressive disorder.

Previous studies have shown that the difference in important structures of the limbic system in TLE patients with depression is significant when compared to non-depressive patients [4–6]. Meanwhile, significant changes in glucose metabolism in their brain regions have been observed in TLE patients with depression. Bromfield et al. [7] did a comparative study of the glucose metabolism of the brain in patients with depression, using ^18^F-2-fluorodeoxyglucose positron emission tomography (FDG-PET), twenty years ago. They found a decrease in glucose metabolism in the frontal region. Gilliam et al. found that the depression was also associated [8] with temporal lobe metabolic decline in temporal lobe epilepsy. Salzberg et al. found that orbital low metabolism was associated [9] with depression using statistical parametric mapping to analyze the FDG-PET images. However, the specific location and significance of abnormal glucose metabolism in the brain regions of TLE patients with depression need more clinical empirical data demonstration. Therefore, this study has included TLE patients with depression and compared them with TLE patients without depression in order to investigate the differences in glucose uptake in the brain regions.

## Materials and methods

### General information

A total of 24 TLE patients treated at the Epilepsy Center of Guangdong 999 Brain Hospital from January 2014 to December 2016, who met the inclusion criteria, were enrolled in this study. The inclusion criteria were as follows: (1) patients who met the diagnostic criteria of the International League Against Epilepsy (ILAE) for TLE; (2) the symptoms of seizure accorded with TLE clinical features, and the video electroencephalogram (VEEG) indicated that the abnormal discharge was confined to the temporal lobe at the interictal stage, and the epileptic discharge at the interictal stage originated from the temporal lobe; (3) patient age ≥ 18 years old; (4) Mini-mental State Examination (MMSE) scores ≥ 24 points; (5) a PET examination showed that the depression was during the interictal period of epileptic seizures, and the HAMD-24 scored ≥ 8 points. The exclusion criteria were as follows: (1) MRI brain tests suggested structural damage outside the temporal lobe; (2) patients with a previous history of mental illness or familial mental illness; and (3) patients with a history of other brain diseases, such as trauma, tumors, intracranial infection, cerebral infarction and cerebral hemorrhage.

All the patients were positive on MRI T1 or T2 imaging. 22 of them showed hippocampal sclerosis and / or amygdala lesions, and 2 patients showed focal malacia, and all patients showed focal cortical dysplasia after operation. The basic clinical data of patients with temporal lobe epilepsy are shown in Additional file 1. There are 11 patients in epilepsy with depression group (7/11 male, 5/11 right TLE), and epilepsy without depression group (9/13 male, 2/13 right TLE). chi-squared test showed that there was no significant difference in gender and lateral distribution of epileptogenic focus between the two groups. Independent sample *t* test there was no significant difference in age, course of disease and the time of taking anticonvulsant drugs between the two groups.

### Methods

#### Scale examination and grouping

The patients were divided into a TLE with depression group (≥ 8) and a TLE without depression group (< 8), according to the HAMD-24 Scale score results. There were 11 patients in the TLE with depression group and 13 patients in the TLE without depression group.

MMSE examination scores > 24 points were recorded for both groups, and they both completed the SCL-90 and MMPI psychometric self-assessment questionnaire and HAMA-21 scale evaluation to make sure they have no other psychotic disorders other than depression.

#### EEG examination

VEEG monitoring: A scalp circular electrode was placed in accordance with the international 10/20 system. The EEG was recorded by single, bipolar lead and sphenoid electrodes. The test time of the EEG was between 8 and 24 h. EEG records included wakefulness, eye opening and closing, flash stimulation, hyperventilation, and sleep induction. The results were read by a neurological electrophysiologist and a neurologist. The spike wave, sharp wave, and spike (sharp)-slow complex wave were all read along with the burst high amplitude slow wave rhythm, spike rhythm, and fast rhythm wave, all of which are associated with epilepsy.

#### ^18^F-FDG PET Brain imaging

All patients underwent ^18^F-FDG PET brain imaging scans at the interictal stage of seizure. The ^18^F-FDG PET developer was provided by Guangzhou High Tech Atom Isotope Medicine Co., Ltd., with a radiochemical purity > 95%. The patients had been fasting for 4–6 h before the examination. Blood glucose was controlled at 4.0–11.1 mmol/L. After being injected with the imaging agent and having lain still for 45–70 min, the patients were given a craniocerebral PET scan at a dose of 3.70–5.55 MBq/kg body weight. The PET/CT imaging system used was the systemic PET/CT (GE Discovery Elite 690, USA). The acquisition conditions were as follows: voltage 140 kV, current 180 mA, scanning layer thickness 2.5 mm, scanning axial field 50 mm, and matrix 512 × 512. The attenuation correction of PET was conducted through CT. The PET images were obtained using an adaptive statistical iterative recombination algorithm.

#### Image processing and analysis

The ^18^F-FDG PET brain images of all patients were analyzed and processed using AW 4.6 processing workstation built-in Cortex ID brain functional metabolism software. Its main procedures are the 3D-SSP method developed by the University of Washington MINOSHIMA et al. [10] as well as correction and conversion techniques that automatically match the standardized brain. These were used to extract a series of predefined surface pixels from the peak metabolic activity in the brain regions. Taking the pontine metabolic activity as a reference baseline, the standardized metabolic activity of the cerebral cortex peak of the patient was fed into the built-in normal group software database so the metabolic activity of the cerebral cortex peak could be compared with that of the normal healthy control group [11, 12]. The Z scores of different parts of the whole brain was obtained, and the Z scores chart of metabolism was shown in the Additional file 1 and Table 1. All the above processes were automatically processed and displayed by the Cortex ID software.

**Table 1:** The basic clinical data of patients with temporal lobe epilepsy

Patient number	Average association SUV Z scores			Averaged cerebral SUV Z scores	Global average SUV Z scores	HAMD-24 total scores
TLE with depression	**Z**right	**Z**left	**Z**right-**Z**left	**Z**averaged cerebral	**Z**global average	
1	− 0.93	− 0.76	− 0.17	− 1.12	− 1.03	11
2	− 0.23	1.2	− 1.43	− 0.24	− 0.27	13
3	− 1.49	− 0.8	− 0.69	− 1.21	− 0.87	16
4	0.8	1.49	− 0.69	0.63	0.57	18
5	0.63	**1.91**	− 1.28	0.58	0.45	26
6	− 0.47	0.08	− 0.55	− 0.45	− 0.36	19
7	0.83	0.18	0.65	0.2	0.12	13
8	0.8	0.37	0.43	0.12	0.05	9
9	− 0.51	− 1.29	0.78	− 1.22	− 1.22	9
10	0.16	− 0.01	0.17	− 0.18	− 0.35	12
11	0.92	0.4	0.52	0.53	0.56	17
Mean	0.05	0.25	− 0.21	− 0.21	− 0.21	14.82
TLE without depression						
12	− 0.75	− 0.44	− 0.31	− 0.59	− 0.45	7
13	0.43	1.39	− 0.96	0.47	0.28	3
14	− 0.66	− 0.87	0.21	− 1.14	− 1.09	6
15	− 0.02	1.08	− 1.1	0.29	0.35	1
16	**1.79**	**3.24**	− **1.45**	**1.99**	**1.65**	7
17	0.74	1.37	− 0.63	0.83	0.68	3
18	− 0.21	− 0.06	− 0.15	− 0.22	− 0.15	5
19	0.66	**1.91**	− 1.25	0.9	0.68	3
20	− 0.1	0.99	− 1.09	0.23	0.12	1
21	0.51	1.28	− 0.77	0.73	0.61	7
22	0.36	0.67	− 0.31	0.35	0.19	6
23	1.07	− 0.04	1.11	0.25	0.39	6
24	1.28	1.12	0.16	0.94	0.83	6
Mean	0.49	0.97	− 0.48	0.47	0.39	4.64

The analysis of uptake difference in the brain regions: Statistical parametric mapping 8 (SPM8) was used to analyze and process the PET/MRI data. (1) MRIcron was used to transform PET image DICOM data into the Analyze7 NIFTI format. (2) The display module in SPM was used to correct the origins of all the patients’ images and rotation direction; (3) A coregister module in SPM was used to register each patient's PET images as 3.0 MRI T1 structural images. (4) A sement module in SPM was used to match each patient’s 3.0 MRI T1 structural images with the SPM built-in standard MRI T1 template. (5) A normalised module in SPM was used to integrate the results obtained in steps 3 and 4 with the patients’ individual standard space PET images. (6) A smooth module in SPM was used for the smoothing of two-fold height and width (FWHM 8 × 8 × 8) for standard space PET images to obtain target images with matrix 79 × 95 × 68 and voxel 2 × 2 × 2. (7) Two groups of images were examined with a voxel-to-voxel independent sample t test (P < 0.01, uncorrected, no less than 30 voxel blocks) to obtain a local brain uptake difference distribution map. (8) Mni2tal tool software was used to locate the different voxels in the naming area of the Brodmann anatomical brain map.

### Statistical analysis

SPSS 21.0 software was used for the statistical analysis of the data results. Measurement data were expressed by M ± SD. The standard uptake value (SUV) Z scores of the same brain regions on the same side of the two groups were tested by independent sample t test. A differential analysis of glucose uptake in the left and right brain regions of each group was performed using a paired t test. P < 0.05 was considered to be a statistically significant difference. In addition, Asymmetry Indices based on 14 specify regional were statistically compared between the two groups. The 14 specify regional including: parietal, temporal, frontal, occipital, posterior, anterior, medial frontal, medial parietal, sensorimotor, visual, caudate nucleus, cerebellum, vermis, lateral average association, which is divided by Brodmann Brain Atlas with Cortex ID workstation software [8–12]. Formula calculation as Z_AI_ = Z_Right_-Z_Left_. So if regional Z_AI_ ≥ 1.64, corresponding to P < 0.05 were considered to have significantly different SUV (Right > Left). If regional Z_AI_ ≤  − 1.64, It means Right < Left. For more details, please refer to the statistical method of Galazzo et al. [13]. The 14 Z_AI_ of each specify regional of the two groups were tested by independent sample t test.

## Results

### The analysis of differential glucose uptake in the brain regions of the two groups

The uptake difference of the brain regions of the TLE with depression group and the TLE without depression group were as follows: left parietal lobe (P = 0.023), right parietal lobe (P = 0.041), left occipital lobe (P = 0.008), right occipital lobe, (P = 0.009), left medial parietal lobe (P = 0.016), right sensorimotor area (P = 0.045), left visual area (P = 0.006), right visual area (P = 0.006), left caudate nucleus (P = 0.039), right caudate nucleus (P = 0.031) and right vermis cerebelli (P = 0.022). The brain regions with a significant margin difference were as follows: the left medial frontal lobe (P = 0.054), the left medial parietal lobe (P = 0.062), the total cerebral cortical mean (P = 0.057) and the total cerebral cortical and subcortical mean (P = 0.051). All the brain regions showed uptake values that were lower in the TLE with depression group than in the TLE without depression group, see Table 2 for details. The Z_AI_ (Asymmetry Indices of Z scores compare to healthy subjects) independent sample t test were no significant difference, see Additional file 1 for details.

**Table 2:** Comparison of difference in glucose uptake (Z scores of SUV) of brain regions between TLE with depression group and TLE without depression group

Brain regions	TLE with depression group (M ± SD)	TLE without depression group (M ± SD)	P
Left parietal lobe	0.001 ± 1.02	0.94 ± 1.11	**0.023**
Right parietal lobe	− 0.31 ± 0.97	0.50 ± 0.88	**0.041**
Left temporal lobe	0.76 ± 1.42	1.10 ± 1.24	0.332
Left frontal lobe	0.10 ± 0.89	0.76 ± 1.02	0.094
Right frontal lobe	− 0.12 ± 0.70	0.34 ± 0.75	0.175
Left occipital lobe	− 1.18 ± 1.08	0.10 ± 1.36	**0.008**
Right occipital lobe	− 1.24 ± 0.83	− 0.24 ± 1.00	**0.009**
Left posterior cingulate gyrus	− 0.12 ± 0.67	0.33 ± 0.69	0.119
Right posterior cingulate gyrus	− 0.39 ± 0.65	− 0.06 ± 0.60	0.231
Left medial frontal lobe	− 0.40 ± 0.80	0.41 ± 1.07	0.054
Right medial frontal lobe	− 0.55 ± 0.75	− 0.003 ± 0.90	0.148
Left medial parietal lobe	0.19 ± 0.90	0.90 ± 1.10	0.062
Right medial parietal lobe	− 0.30 ± 0.90	0.76 ± 0.91	**0.016**
Left sensorimotor area	− 0.005 ± 0.79	0.59 ± 0.82	0.106
Right sensorimotor area	− 0.37 ± 0.86	0.38 ± 0.70	**0.045**
Left visual area	− 1.97 ± 1.40	− 0.43 ± 1.31	**0.006**
Right visual area	− 1.98 ± 1.28	− 0.60 ± 1.23	**0.006**
Left caudate nucleus	− 1.35 ± 1.10	− 0.39 ± 1.12	**0.039**
Right caudate nucleus	− 1.18 ± 1.54	− 0.11 ± 0.75	**0.031**
Right cerebellar vermis	− 0.78 ± 0.71	− 0.08 ± 0.66	**0.022**
Left joint mean	0.30 ± 1.01	0.90 ± 1.08	0.117
Right joint mean	− 0.001 ± 0.79	0.39 ± 0.74	0.289
Total cerebral cortex mean	− 0.21 ± 0.71	0.39 ± 0.77	0.057
Total cortical and subcortical mean	− 0.21 ± 0.63	0.31 ± 0.66	0.051

### An analysis of the left–right pairing of glucose uptake in the brain regions of the two groups

The TLE with depression group: The uptake values of the left posterior cingulate cortex, medial parietal lobe and sensorimotor area were significantly higher than those of the right posterior cingulate gyrus, medial parietal lobe and sensorimotor area (P < 0.05). See Table 3 for details.

**Table 3:** Left and right paired samples t test results of uptake difference (Z scores of SUV) in TLE with depression group

Brain regions	Left (M ± SD) vs. right (M ± SD)	t	P
Left posterior cingulate gyrus vs. Right posterior cingulate gyrus	− 0.12 ± 0.67 vs. − 0.39 ± 0.65	4.363	0.001
Left medial parietal lobe vs. Right medial parietal lobe	0.19 ± 0.90 vs.− 0.26 ± 0.90	2.741	0.021
Left sensorimotor area vs. Right sensorimotor area	− 0.01 ± 0.79 vs. − 0.37 ± 0.86	2.975	0.014

The TLE without depression group: the mean uptake values of the left parietal lobe, temporal lobe, frontal lobe, occipital lobe, posterior cingulate gyrus, and medial frontal lobe and the joint mean were significantly higher than those of the right parietal lobe, temporal lobe, frontal lobe, occipital lobe, posterior cingulate gyrus, medial frontal lobe and joint mean (P < 0.05). The uptake values of the left cerebellum were significantly lower than those of the right cerebellum (P = 0.038). See Table 4 for details.

**Table 4:** Left and right paired samples t test results of uptake difference (Z scores of SUV) in TLE without depression group

Brain regions	Left (M ± SD) vs. Right (M ± SD)	*t*	P
Left parietal lobe vs. Right parietal lobe	0.94 ± 1.11 vs. 0.50 ± .87647	2.339	0.037
Left temporal lobe vs. Right temporal lobe	1.10 ± 1.24 vs. 0.44 ± 0.73	2.279	0.042
Left frontal lobe vs. Right frontal lobe	0.76 ± 1.02 vs. 0.34 ± 0.75	2.622	0.022
Left occipital lobe vs. Right occipital lobe	0.10 ± 1.36 vs. − 0.24 ± 1.00	2.296	0.040
Left posterior cingulate gyrus vs. Right posterior cingulate gyrus	0.33 ± 0.69 vs. − 0.06 ± 0.60	3.767	0.003
Left medial frontal lobe vs. Right medial frontal lobe	0.41 ± 1.07 vs. − 0.003 ± 0.90	2.590	0.024
Left cerebellum vs. Right cerebellum	− 0.08 ± 0.76 vs. 0.08 ± 0.83	− 2.337	0.038
Left joint mean vs. Right joint mean	0.90 ± 1.08 vs. 0.39 ± 0.74	2.523	0.027

### The extraction results of glucose metabolism uptake values in the brain regions of the TLE with depression group and the TLE without depression group

**Fig. 1:**
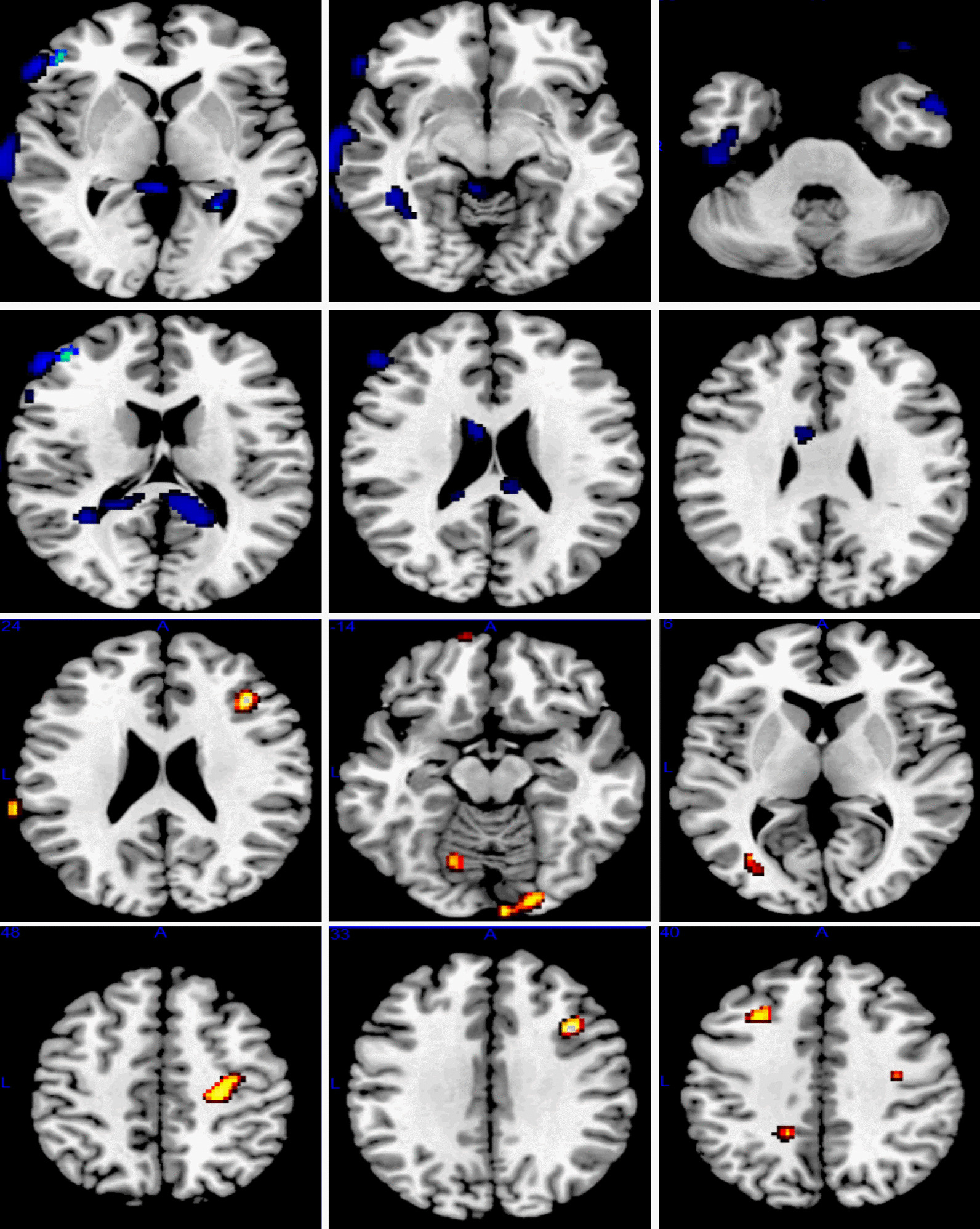
Difference in glucose metabolism uptake values of cerebral regions between the TLE with depression group and TLE without depression group. Blue block: Metabolic regions of TLE with depression group < TLE without depression group; Red block: Metabolic regions of TLE with depression group > TLE without depression group

An SPM8 Result module was used to compare the TLE with depression group and the TLE without depression group with respect to voxel-based glucose metabolic uptake values extracted in the brain regions. The threshold standard was uncorrected (P < 0.01). The voxel mass was not less than 30. The results showed that the SUV in the brain regions of the top of the inferior frontal gyrus of the left anterior frontal lobe, left inferior frontal gyrus, left temporal lobe and left fusiform gyrus in the TLE with depression group was lower than that of the TLE without depression group. The SUV in the brain regions of the left inferior frontal gyrus, right visual union cortex, right superior posterior cingulate cortex, right anterior frontal lobe, and right secondary somatosensory cortex in the TLE with depression group was higher than in the TLE without depression group. See Table 5 and Fig. 1 for details.

**Table 5:** Summary of difference in glucose metabolic uptake values in brain regions between epilepsy with depression group and epilepsy without depression group

Brain regions	x	y	z	Number of voxels	Z value	P
TLE with depression < TLE without depression						
Top of inferior frontal gyrus of left inferior frontal lobe	44	48	14	463	3.62	< 0.001
Left inferior frontal gyrus	42	− 12	− 42	154	3.16	< 0.001
Left temporal lobe	62	− 2	− 10	778	3.03	0.001
Left fusiform gyrus	64	− 40	− 16	82	2.95	0.002
TLE with depression > TLE without depression						
Left inferior frontal gyrus	42	14	30	118	3.37	< 0.001
Right visual joint cortex	− 28	− 80	12	78	2.69	0.004
Right visual joint cortex	− 16	− 72	− 10	51	2.67	0.004
Right superior posterior - cingulate cortex	− 12	− 48	38	38	2.67	0.004
Right prefrontal lobe	− 24	20	42	39	2.62	0.004
Right secondary somato- sensory cortex	− 64	− 38	26	49	2.58	0.005

## Discussion

Henry et al. [14], studied ^18^F FDG-PET and found that most patients with complex partial seizures originating from a unilateral temporal lobe had temporal lobe hypometabolism at the interictal stage. This could extend to the ipsilateral external temporal region. The metabolism of the ipsilateral frontal lobe, parietal lobe and basal ganglia also decreased, but not as significantly as that of the temporal lobe and thalamus. An FDG-PET SPM analysis showed that 79% of the TLE patients (27/34) had hypometabolism [15, 16] in the ipsilateral temporal lobe. A paired t test confirmed that the metabolic activity of the ipsilateral hemisphere decreased when compared to the activity of the contralateral hemisphere. A Voxel-based analysis supported a decrease in the glucose metabolism rate along with prolonged duration of epilepsy in the temporal cortex and other adjacent cortical regions. The relative increase in glucose metabolism in the contralateral temporal lobe suggested that there may have been compensatory changes in the hemisphere [17]. In addition, Wunderlich et al. [18], differentiated between and compared the brain glucose metabolism and depression symptoms of lateral temporal lobe epilepsy and medial temporal lobe epilepsy. They found that the glucose metabolism patterns of the two groups decreased significantly when compared to the control group. By comparing and analyzing glucose metabolism in the homologous anatomical regions of each side, 35% of epileptic patients with an attack originating in the lateral temporal lobe had the largest regional depression, while 56% of epileptic patients with the attack originating in the medial temporal lobe had a moderate decrease in glucose metabolism in the ipsilateral medial temporal region (up to a 20% difference). No regions with increased glucose metabolism were found in either group. Victoroff et al. [19], have observed the frequency of major depressive episodes (MDEs) and the decreased metabolism in left and right temporal lobes in patients with left and right seizures. Of the 52 patients, 33 (62%) had a history of interictal depression, of which 16 (30%) met the criteria for one or more severe depressive episodes. The patients with mostly left-sided seizures had a history of depression. The PET scan showed that 36 patients had definite unilateral temporal lobe hypometabolism. Most patients with left temporal lobe hypometabolism had a history of severe depression. The combination of left temporal lobe hypometabolism and severe hypometabolism was closely related to a history of severe depression. In addition, more severe depressive episodes tended to occur in those patients with right temporal lobe hypometabolites [20]. Therefore, the laterality of the attack and the degree of hypometabolism of the temporal lobe during an attack may be the interdependent factor leading to the risk of depressive disorder in patients with complex partial epilepsy.

This present study found that the hypometabolic regions of the TLE with depression group are mainly located in the right frontal lobe, temporal lobe and fusiform gyrus compared with the TLE without depression group, while the hypermetabolic regions of the TLE with depression group are mainly found in the left frontal lobe, visual joint cortex, superior posterior cingulate cortex and secondary somatosensory cortex compared with the TLE without depression group. A possible reason for this is that the proportion (5/11) of epileptic regions originating in the right brain region in the TLE with depression group is higher than that in the TLE without depression group (2/13). The other reason is that right temporal lobe epilepsy is more likely to lead to low metabolic epileptic depression. Previous studies have suggested that left epileptic foci are more likely to cause depressive symptoms [21, 22], while some studies have suggested that right epileptic foci are more likely to cause depressive symptoms [20]. Yet other studies have suggested that epileptic depression is not significantly correlated with a particular side [23]. Moreover, a postoperative study discovered that the severity of depressive symptoms in patients undergoing epilepsy surgery was associated with the extent of hippocampal and amygdala resection. This correlation was more pronounced in the left resection [24]. In this study, our SPM SUV Voxel-to-Voxel analysis showed the top of the inferior frontal gyrus of the left anterior frontal lobe, left inferior frontal gyrus, left temporal lobe and left fusiform gyrus in the TLE with depression group was lower than that of the TLE without depression group. While left inferior frontal gyrus, right visual union cortex, right superior posterior cingulate cortex, right anterior frontal lobe, and right secondary somatosensory cortex in the TLE with depression group was higher than in the TLE without depression group. The higher degree of left hypometabolism can support the pathological mechanism of epileptic depression, This is consistent with the evidence of Kwon and Shamim et al. [8, 21, 22] which may be related to the fact that left temporal lobe epilepsy is more likely to cause cognitive impairment [25].

On the mechanism of glucose metabolism of neurons, Crane et al. [26] Considered that neurons in the low metabolism area could not make effective neural connection, and low metabolism occurred due to the damage of cell function caused by epileptogenic foci discharge. Due to repeated focal seizures, temporal lobe neurons showed functional changes, neuronal electrical activity decreased, resulting in decreased glucose metabolism and low metabolism. The study of Koutmumanidis et al. [27] And Altay et al. [28] also showed that the low activity area of EEG was highly correlated with the low metabolism area of temporal lobe, but not with the pathological state of medial temporal lobe sclerosis. This study seems to support those of Crane’s viewpoint. Other fMRI studies of TLE with depression have also confirmed that depression patients have lower neuronal connectivity centrality, which tends to be more likely to cause depression [29, 30].

However, we should be cautious about these inconsistent study conclusions. After all, the sample sizes are small and there are unevenly matched samples, so more equally matched samples are needed to validate these conclusions in the future.

## Conclusions

This study has found that TLE patients with depression and TLE patients without depression have uptake differences in ^18^F-FDG in multiple brain regions. The hypometabolic regions in the TLE with depression group are mainly located in the left frontal lobe, temporal lobe and fusiform gyrus when compared to the TLE without depression group. The hypermetabolic regions of the TLE with depression group are mainly found in the right frontal lobe, visual joint cortex, superior posterior cingulate cortex and secondary somatosensory cortex. The TLE without depression group has a significant glucose hypermetabolic compensatory mechanism on the right side. In addition, both the TLE with depression group and the TLE without depression group have a high metabolic compensation in the non-epileptic area during the interictal period.

## Supplementary information

**Additional file 1.** Basic clinical data and SUV Z scores of patients with temporal lobe epilepsy.

## Data Availability

The datasets used and/or analysed during the current study available from the corresponding author on reasonable request.

## Abbreviations

TLE: Temporal lobe epilepsy
FDG-PET: 18F-2-fluorodeoxyglucose positron emission tomography
ILAE: International League Against Epilepsy Epileptic
MMSE: Mini-mental State Examination
SUV: Standard uptake value
SPM8: Statistical parametric mapping 8
MDEs: Major depressive episodes
